# Effect of Surgical Masks on Cardiopulmonary Function in Healthy Young Subjects: A Crossover Study

**DOI:** 10.3389/fphys.2021.710573

**Published:** 2021-09-10

**Authors:** Guolin Zhang, Mei Li, Meifeng Zheng, Xiaoqing Cai, Jinyu Yang, Shengqing Zhang, Anniwaer Yilifate, Yuxin Zheng, Qiang Lin, Junjie Liang, Lan Guo, Haining Ou

**Affiliations:** ^1^Department of Cardiac Rehabilitation, Guangdong Provincial People’s Hospital, Guangzhou, China; ^2^Department of Rehabilitation Medicine, The Fifth Affiliated Hospital of Guangzhou Medical University, Guangzhou, China; ^3^Department of Rehabilitation Medicine, Guangzhou Medical University, Guangzhou, China; ^4^Guangzhou Key Laboratory of Enhanced Recovery After Abdominal Surgery, The Fifth Affiliated Hospital of Guangzhou Medical University, Guangzhou, China

**Keywords:** surgical masks, cardiopulmonary fitness, exercise endurance, ventilation, end-tidal carbon dioxide partial pressure

## Abstract

**Objective:** Mask plays an important role in preventing infectious respiratory diseases. The influence of wearing masks in physical exercise on the human body needs to be studied. The purpose of this study is to explore the influence of wearing surgical masks on the cardiopulmonary function of healthy people during exercise.

**Methods:** The physiological responses of 71 healthy subjects (35 men and 36 women, age 27.77 ± 7.76 years) to exercises with and without surgical masks (mask-on and mask-off) were analyzed. Cardiopulmonary function and metabolic reaction were measured by the cardiopulmonary exercise test (CPET). All tests were carried out in random sequence and should be completed in 1 week.

**Results:** The CPETs with the mask-on condition were performed undesirably (*p* < 0.05), and the Borg scale was higher than the mask-off (*p* < 0.001). Rest oxygen uptake ($\dot{\text{V}}{\text{O}}_{2}$) and carbon dioxide production ($\dot{\text{V}}$CO_2_) with the mask-on condition were lower than mask-off (*p* < 0.01), which were more obvious at peak exercise ($\dot{\text{V}}$O_2__*peak*_: 1454.8 ± 418.9 vs. 1628.6 ± 447.2 ml/min, *p* < 0.001; $\dot{\text{V}}$CO_2__*peak*_: 1873.0 ± 578.7 vs. 2169.9 ± 627.8 ml/min, *p* = 0.005), and the anaerobic threshold (AT) brought forward (*p* < 0.001). At different stages of CPET with the mask-on condition, inspiratory and expiratory time (Te) was longer (*p* < 0.05), and respiratory frequency (Rf) and minute ventilation ($\dot{\text{V}}$_*E*_) were shorter than mask-off, especially at peak exercise (Rf_*peak*_: 33.8 ± 7.98 vs. 37.91 ± 6.72 b/min, *p* < 0.001; $\dot{\text{V}}$_*Epeak*_: 55.07 ± 17.28 vs. 66.46 ± 17.93 l/min, *p* < 0.001). *V*_*T*_ was significantly lower than mask-off just at peak exercise (1.66 ± 0.45 vs. 1.79 ± 0.5 l, *p* < 0.001). End-tidal oxygen partial pressure (PetO_2_), end-tidal carbon dioxide partial pressure (PetCO_2_), oxygen ventilation equivalent ($\dot{\text{V}}$_*E*_/$\dot{\text{V}}$O_2_), and carbon dioxide ventilation equivalent ($\dot{\text{V}}$_*E*_/$\dot{\text{V}}$CO_2_) with mask-on, which reflected pulmonary ventilation efficiency, were significantly different from mask-off at different stages of CPET (*p* < 0.05), but no significant difference in percutaneous oxygen saturation (SpO_2_) was found. Differences in oxygen pulse ($\dot{\text{V}}$O_2_/HR), oxygen uptake efficiency slope (OUES), work efficiency (△$\dot{\text{V}}$O_2_/△W), peak heart rate (HR), and peak systolic blood pressure (BP) existed between two conditions (*p* < 0.05).

**Conclusion:** Wearing surgical masks during aerobic exercise showed certain negative impacts on cardiopulmonary function, especially during high-intensity exercise in healthy young subjects. These results provide an important recommendation for wearing a mask at a pandemic during exercises of varying intensity. Future research should focus on the response of wearing masks in patients with related cardiopulmonary diseases.

## Introduction

In 2020, a new type of coronavirus swept the world, which was highly contagious and harmful, seriously endangering human health, even causing death and affecting social stability. At present, there has been no specific drug to treat the COVID-19 virus, so it is very important to actively prevent its transmission (Indu et al., 2020). The main modes of transmission include airborne transmission, aerosol transmission, and contact transmission (Fink et al., 2020). The role of the mask in other respiratory diseases has been confirmed (Liang et al., 2020). There has been also evidence that masks could help to prevent the spread of COVID-19 (Chan et al., 2020; Tirupathi et al., 2020). A study in the United States showed that if 80% of the people in New York wore a mask with moderate efficiency (50%), the peak daily mortality could have been reduced by 34–58%. Even wearing masks with low efficiency (20%) in low-risk areas still had a certain effect, which could have been reduced community transmission in COVID-19 (Eikenberry et al., 2020). Therefore, on April 3, 2020, the US Centers for Disease Control and Prevention (CDC) recommended that the public should wear a mask when going out (Islam et al., 2020). Although the marketing of vaccines is conducive to the control of transmission in COVID-19, the daily protection work cannot be ignored. In this year, the mask has become an essential part of daily life and work.

At present, most research studies on the effects of wearing masks on human physiology have addressed the safety and performance of mask before it was put on the market. Some research suggested that wearing masks could impact human physiological functions, especially cardiopulmonary function, such as increased airway resistance, hypoxia, carbon dioxide retention, and other lung function changes, leading to increased heart load, insufficient coronary perfusion, decreased muscle aerobic metabolism, increased anaerobic metabolism, and even affecting renal function and immune function (Lee and Wang de, 2011; Chandrasekaran and Fernandes, 2020). Wearing masks will also increase temperature, humidity, and discomfort on the face (Scarano et al., 2020), and affect exercise performance (Driver et al., 2021). The type of mask, the time of wearing the mask, the type and intensity of the activity, and the environment will have different effects. The limited small sample study indicated that it was relatively safe to wear surgical masks or filter masks (such as N95) for daily activities and short-term low-intensity exercise (Goh et al., 2019). There were few reports on the physiological changes of the human body when wearing masks for moderate and high-intensity exercise. Research on 16 athletes demonstrated that after wearing masks, the maximum oxygen uptake ($\dot{\text{V}}$O_2_) and ventilation decreased, and 11 athletes had acute dyspnea at the peak of maximal exercise test (Egger et al., 2021). Athletes with good cardiopulmonary reserve function had such changes, not to mention ordinary healthy people. Fikenzer et al. (2020) conducted cardiopulmonary exercise tests (CPETs) on 12 healthy men under different masks. The results showed that surgical masks and FFP2/N95 masks could reduce ventilation, exercise endurance, and comfort. Lässing et al. (2020) tested 14 healthy men with constant power exercise and found out that the peak heart rate (HR) and cardiac output (CO) were larger when wearing surgical masks, but there were no differences in the changes in blood pressure (BP) and blood lactate. However, the research of Shaw K demonstrated that wearing masks for strenuous exercise had no obvious effect on the exercise performance of healthy young people, such as percutaneous oxygen saturation (SpO_2_), tissue oxygenation index, exercise maximum load, exercise HR, and rating of perceived exertion (RPE) (Shaw et al., 2020). A study on sarcopenia patients also showed that when wearing surgical or FFP2 masks for resistance training the changes in HR, HR variability, blood lactic acid concentration, self-perceived fatigue, and muscle strength were similar with not wearing masks (Ramos-Campo et al., 2021).

The influence of masks on human cardiopulmonary function during exercise remains unclear, and proper exercise training is an effective measure to prevent diseases and improve the prognosis of diseases. In the ongoing epidemic, people still need to wear masks for a long time, so it is necessary to further study the influence of wearing masks on human cardiopulmonary function. Therefore, this study intends to explore the effect of surgical masks in healthy people by monitoring the changes in cardiopulmonary function and metabolic parameters to guide safe exercise while wearing surgical masks.

## Materials and Methods

### Subjects

The research is a self-controlled trial, which has been approved by the Ethics Committee of Guangdong Provincial People’s Hospital (ethics number: GDREC2020145H) and can be consulted in the Chinese Clinical trial registry (No. ChiCTR2000033449). Healthy subjects were recruited from June 1, 2020 to July 31, 2020. The inclusion criteria of the study included subjects between 18 and 40 years old, who participated voluntarily, passed PAR-Q questionnaire screening, had normal rest electrocardiogram and static lung function, and had signed informed consent. The presence of any of the following conditions would not be allowed to participate in the study, including the history of COVID-19 infection, previous cardiopulmonary diseases (such as asthma, chronic bronchitis, pulmonary fibrosis, emphysema, and congenital heart disease), situations (such as exercise asthma and epilepsy) that may deteriorate due to exercise, physical disability caused by articulations or neuromuscular diseases, lower respiratory tract infection in the past 2 weeks, acute upper respiratory tract infection or symptomatic rhinitis in the past 1 week, mental or cognitive disabilities, smoking, pregnancy, menstrual period and lactation period, and other contraindications of CPET.

### Study Design

**FIGURE 1:**
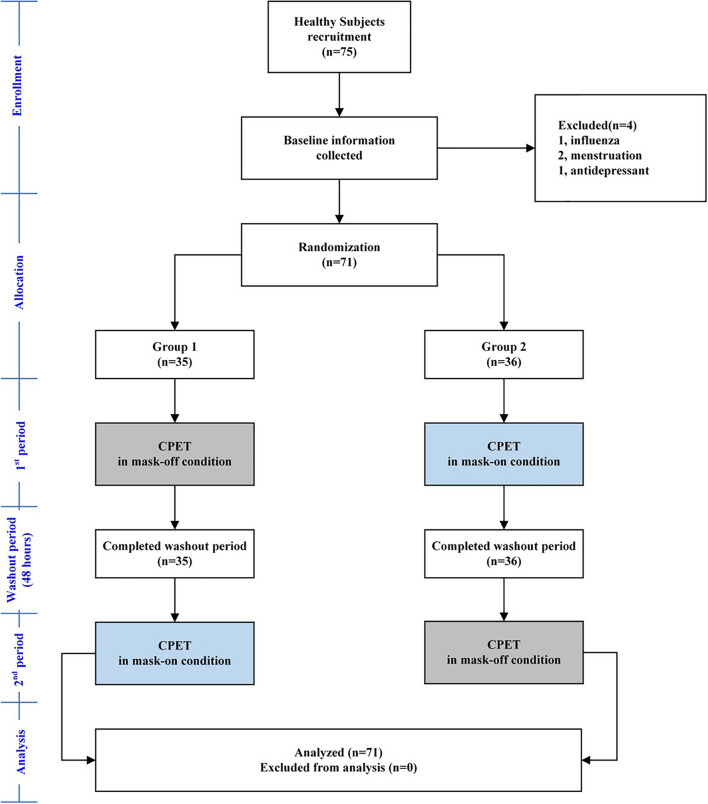
The study flow diagram.

Every subject was asked to go to the hospital to complete the CPET two times. The two tests were carried out under conditions A (mask-on) and B (mask-off). The interval between the two tests was at least 24 h and completed within 1 week. Every subject was randomly assigned to CPET according to AB sequence or BA sequence (flow chart is as Figure 1). All CPETs were completed by one professional. When subjects entered the experiment, medical data were collected, and vital signs, height, weight, rest electrocardiogram, and static lung function tests were carried out by special personnel.

### Masks

**FIGURE 2:**
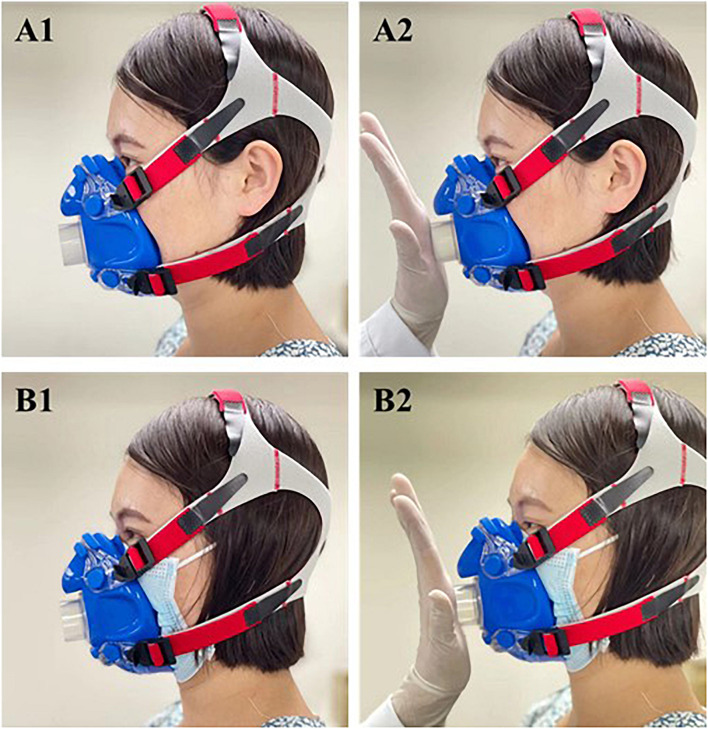
Fit comparison of mask-on and mask-off condition. **(A1)** Mask-off condition. **(A2)** Fit test in mask-off condition. **(B1)** Mask-on condition. **(B2)** Fit test in mask-on condition.

All subjects were provided with typical and widely used disposable ear-hanging surgical masks for adults (Guangzhou Tianhe Haozheng Sanitary Materials Factory, Guangzhou, China). The spirometry mask (V2 MASK, United States) used in CPET was selected according to the face shape of subjects and required to wear comfortably. The spirometry mask was placed over the surgical masks and fixed with head straps in a leak-proof manner (Figure 2). Before each CPET, an air leakage test was conducted to confirm whether the mask fits correctly. The tester completely blocked the ventilation valve of the spirometry mask with the palm of the hand. Then, the subjects breathed with maximal force against the mask to check for leaks (Figure 2). This maneuver was repeated until no acoustic, visual, and sensory indications of leakage were detectable.

### Testing Equipment

#### Cardiopulmonary Exercise Test

The CPET was performed with JAEGER Master Screen CPX (Germany) system and the cycle ergometer (Ergoline 150P, Germany). Calibration of the gas analyzer was performed before each test, including flow sensor calibration, indoor air calibration, standard balance gas (using 16%O_2_, 5%CO_2_, and N_2_) calibration, and delay calibration. Subjects were asked to take more than 2 h after eating without satiety, were advised to avoid eating foods containing caffeine and alcohol at least 12 h before the test, and were ensured to take adequate rest the day before the test.

### Cardiopulmonary Exercise Test Protocol

The CPETs were performed using a ramped cycle ergometer protocol, and incremental power was calculated to let subjects finish the exercise load test in 8–12 min. The incremental power selection was calculated with the following formulas (Costa et al., 2015):


V.O(ml/min)2withoutload=150+(6×bodyweightkg)V.Omax2(ml/min)=(heightcm-ageY)×20(male),V.Omax2(ml/min)=(heightcm-ageY)×14(female)Incrementalpowerperminute=(V.Omax2-V.Owithout2load)/100.

### Specific Process

First, the forced vital capacity (FVC), forced expiratory volume in one second (FEV_1_), FEV_1_/FVC, and maximum minute ventilation (MVV) were measured. Then, while the subject was wearing a spirometry mask connected with a gas analyzer, a 12-leads ECG was connected, BP cuff and fingertip oxyhemoglobin saturation meter were adjusted, and the seat height of the cycle ergometer was set. Next, the subjects rested for 2 min in a sitting position, collecting rest real-time gas metabolism and ECG data. After 2-min warming up with 0 W, the subjects pedaled the cycle according to the set incremental power (15–25 W/min) until exhaustion, and the pedaling speed was kept at about 60 RPM. The test system automatically collected real-time gas metabolism data through the gas analyzer, ECG, and BP data through telemetry cardiogram monitor, and automatically calculated average values every 10 s of parameters that reflect metabolism, gas exchange, ventilation efficiency, and cardiovascular function, according to the oxygen concentration, carbon dioxide concentration, and respiratory flow rate measured breath-by-breath. When the subjects reached the best effort standard (Dougherty et al., 2018), which met at least three of the following items: (1) RPE ≥ 17; (2) respiratory exchange rate (RER) ≥ 1.10; (3) HR_*peak*_ reaches more than 90% of the predicted maximum HR; (4) $\dot{\text{V}}$O_2_ increases <200 ml (as increased power) or reached other criteria for terminating exercise test, the subjects pedaled for 3 min at 0 W, resting in the sitting position for 3 min to finish the test.

### Criteria for Terminating the Exercise Test

The test can be terminated when any of the following items are met:

1. Pedal to exhaustion (RPE ≥ 17–18), the pedaling speed cannot be maintained, lower than 40 RPM.
2. Clinical symptoms: fatigue or dyspnea, severe chest pain; systolic BP decreased by 10 mmHg; cerebral ischemic symptoms, such as dizziness and headache; poor peripheral circulation, such as the face is pale and BP cannot be measured; the subject asked to stop strongly.
3. ECG changes: exercise-induced ST-segment depression ≥ 3 mm or ST-segment elevation > 1 mm; HR did not increase but decreased as exercise intensity increased; the ECG axis was extremely offset; ventricular tachycardia; supraventricular tachycardia; frequent ventricular extrasystoles caused or aggravated by exercise; indoor conduction block caused by exercise.
4. Metabolic index: RER was above 1.15; SpO_2_ dropped below 86%, and respiratory rate was more than 50 beats/min. Oxygen pulse and $\dot{\text{V}}$O_2_ appeared to plateau or decrease. HR reserve (HRR) and breathing reserve (BR%) were exhausted.

### Rating of Perceived Exertion and Borg Dyspnea Scale

After each CPET, the subjects were asked about the degree of discomfort or intolerance using RPE and Borg dyspnea scale. RPE was scored from 6 to 20, indicating extremely light to exhaustion. Borg dyspnea scale was from 0 to 10, indicating no dyspnea at all to extremely severe dyspnea.

### Outcomes

This study obtained data of the following parameters:

(1) CPET performance: CPET test duration, maximum power, RPE score, and Borg *dyspnea* scale.
(2) Parameters reflecting metabolism: $\dot{\text{V}}$O_2_, carbon dioxide production ($\dot{\text{V}}$CO_2_), metabolic equivalent (MET), RER, and percentage of oxygen uptake at anaerobic threshold (AT) in predicted maximal oxygen uptake ($\dot{\text{V}}$O_2_@AT/$\dot{\text{V}}$O_2_max pre %). Among them, AT was determined by the V-slope method.
(3) Parameters reflecting lung ventilation and ventilation efficiency: inspiratory time (Ti), expiratory time (Te), respiratory frequency (Rf), tidal volume (V_*T*_), minute ventilation volume ($\dot{\text{V}}$_*E*_), end-tidal oxygen partial pressure (PetO_2_), end-tidal carbon dioxide partial pressure (PetCO_2_), oxygen ventilation equivalent ($\dot{\text{V}}$_*E*_/$\dot{\text{V}}$O_2_), and carbon dioxide ventilation equivalent ($\dot{\text{V}}$_*E*_/$\dot{\text{V}}$CO_2_), carbon dioxide ventilation equivalent slope ($\dot{\text{V}}$_*E*_/$\dot{\text{V}}$CO_2_ Slope), and BR% and SpO_2_.
(4) Parameters reflecting cardiovascular function: oxygen pulse ($\dot{\text{V}}$O_2_/HR), work efficiency (△$\dot{\text{V}}$O_2_/△W), oxygen uptake efficiency slope (OUES), BP, HR, and HRR.

The main outcome measures were $\dot{\text{V}}$_*E*_, Vt, PetCO_2_, $\dot{\text{V}}$O_2_, $\dot{\text{V}}$O_2_/kg, $\dot{\text{V}}$CO_2_, $\dot{\text{V}}$O_2_/HR, and OUES.

### Sample Size Estimation

According to the pre-experiment results, the main outcome measure was $\dot{\text{V}}$O_2__*peak*_/kg, and the software G^∗^Power 3.1.92 was used. The authors assumed that the risk was 0.05 and the risk was 0.95, the average difference of $\dot{\text{V}}$O_2__*peak*_/kg between two groups of CPETs (with and without masks) was 11.7 (SD is 13.27) ml/min/kg. The results showed that 44 subjects were needed.

### Statistical Analysis

SPSS 25.0 statistical software was used for data processing. The measurement data were tested for normality, and the normal distribution data were expressed in mean ± SD ($\bar{x}$ ± s) and the non-normal distribution data was expressed in the median; enumeration data used cases and the rate (%). Paired *t*-test was used for self-comparison of normal distribution variables; the Wilcoxon rank-sum test was used for self-comparison of non-normal distribution variables. The significance level was set at *p* < 0.05.

## Results

### Baseline Characteristic

A total of 75 subjects were recruited, in which 4 subjects were excluded because 1 had influenza, 2 were menstruating, and 1 was under antidepressant. So, 71 healthy subjects (men 35, women 36) were recruited through outpatient service, with an average age of 27.77 ± 7.76 years and an average BMI of 21.46 ± 2.75 kg/m^2^. Among them, 77% of subjects exercised less than three times per week and 1 h per day. The mean FVC, FEV_1_, FEV_1_/FVC, and MVV of subjects were 3.89 ± 0.78 l, 3.27 ± 0.60 l, 84.62 ± 6.72%, and 118.40 ± 30.10 l, respectively (Table 1).

**TABLE 1:** Baseline characteristics.

Items	Unit	Mean ± SD
Age	Years	27.8 ± 7.76
**Gender**		
Male		35
Female		36
Weight	kg	59.6 ± 10.6
Height	cm	166.3 ± 8.50
BMI	kg/m^2^	21.5 ± 2.75
**Exercise frequency**		
Number of subjects (≥3 times/week, 1 h/day)		16
Number of subjects (<3 times/week, 1 h/day)		55
**Static lung function**		
FVC	l	3.89 ± 0.78
FEV_1_	l	3.27 ± 0.60
FEV_1_/FVC	%	84.6 ± 6.72
MVV	l/min	118.4 ± 30.1

*BMI, body mass index; FVC, forced vital capacity; FEV_1_, forced capacity volume in the first second; MVV, maximal voluntary ventilation; kg, kilogram; cm, centimeter; m, meter; min, minute; l, liter; SD, standard deviation.*

### Cardiopulmonary Exercise Test Performances

The CPET test time was slightly shorter in mask-on condition than mask-off (7.97 ± 1.50 vs. 8.20 ± 1.39 min, *p* = 0.052), and the maximum power in mask-on condition was also significantly lower than mask-off (142.9 ± 44.22 vs. 149.8 ± 46.04 W, *p* < 0.001). The RPE and the Borg scale of the two conditions were significantly different (*p* < 0.001), and the Borg scales of the mask-on condition were higher (5.69 ± 1.62 vs. 4.78 ± 1.72, *p* < 0.001) (Table 2).

**TABLE 2:** The comparison of cardiopulmonary exercise performance between mask-on and mask-off condition.

	Unit	Mask-off	Mask-on	Cohen’s *d* effect size	*P* value
Test period	Minutes	8.20 ± 1.39	7.98 ± 1.50	0.16	0.052
Maximum load	Watt	149.8 ± 46.0	142.9 ± 44.2	0.15	**<0.001**
Borg scale	Scores	4.78 ± 1.72	5.69 ± 1.62	0.54	**<0.001**

*The values were shown in mean ± standard deviation. Significance level was set at *P* < 0.05. Significant results are indicated in bold.*

### Metabolic Reaction Parameters

The results of metabolic reaction parameters showed that $\dot{\text{V}}$O_2_, $\dot{\text{V}}$O_2_/kg, and MET of mask-off and mask-on were significantly different in each stage of CPET (*p* < 0.001), and $\dot{\text{V}}$O_2__*peak*_ and $\dot{\text{V}}$O_2__*peak*_/kg of mask-on was significantly lower than mask-off ($\dot{\text{V}}$O_2__*peak*_: 1454.8 ± 418.9 vs. 1628.6 ± 447.2 ml/min, *p* < 0.001; $\dot{\text{V}}$O_2__*peak*_/kg: 24.33 ± 4.96 vs. 27.3 ± 5.47 ml/min/kg, *p* < 0.001). There were significant differences in both conditions on $\dot{\text{V}}$O_2_@AT/$\dot{\text{V}}$O_2_ max pre % (*p* < 0.001). The $\dot{\text{V}}$O_2_ of mask-on was lower than mask-off in the rest period of CPET (209.7 ± 81.74 vs. 250.2 ± 94.14 ml/min, *p* = 0.007), and the difference was more significant in the peak exercise period (1873.0 ± 578.7 vs. 2169.9 ± 627.8 ml/min, *p* = 0.005). There were significant differences in both conditions on RER only at peak exercise (*p* < 0.001) (Table 3).

**TABLE 3:** The comparison of metabolic reaction parameters between mask-on and mask-off condition.

	Mask-off	Mask-on	*P* value
**$\dot{\text{V}}$O_2_ (ml/min)**			
Rest	288.2 ± 98.03	241.0 ± 90.3	**<0.001**
AT	1024.2 ± 268.0	936.9 ± 314.9	**<0.001**
Peak	1628.6 ± 447.2	1454.8 ± 418.9	**<0.001**
**$\dot{\text{V}}$O_2_@AT/$\dot{\text{V}}$O_2_max pre %**	44.7 ± 8.25	40.7 ± 9.20	**<0.001**
**$\dot{\text{V}}$O_2_/kg (ml/min/kg)**			
Rest	4.88 ± 1.53	4.08 ± 1.44	**<0.001**
AT	17.4 ± 4.09	15.8 ± 4.39	**<0.001**
Peak	27.3 ± 5.47	24.3 ± 4.96	**<0.001**
**$\dot{\text{V}}$CO_2_ (ml/min)**			
Rest	250.2 ± 94.1	209.7 ± 81.7	**0.007**
AT	1020.7 ± 273.8	925.4 ± 357.3	**0.018**
Peak	2169.9 ± 627.8	1873.0 ± 578.7	**0.005**
**MET**			
Rest	1.40 ± 0.45	1.16 ± 0.41	**<0.001**
AT	4.96 ± 1.17	4.50 ± 1.25	**<0.001**
Peak	7.80 ± 1.58	6.97 ± 1.43	**<0.001**
**RER**			
Rest	0.86 ± 0.12	0.88 ± 0.10	0.220
AT	0.99 ± 0.08	0.98 ± 0.08	0.353
Peak	1.32 ± 0.13	1.27 ± 0.13	**<0.001**

*The values were shown in mean ± standard deviation. Significance level was set at *P* < 0.05. $\dot{\text{V}}$O_2_ oxygen uptake; AT, anaerobic threshold; $\dot{\text{V}}$CO_2_, carbon dioxide production; MET, metabolic equivalent; RER, respiratory exchange ratio. Significant results are indicated in bold.*

### Lung Function Parameters

**FIGURE 3:**
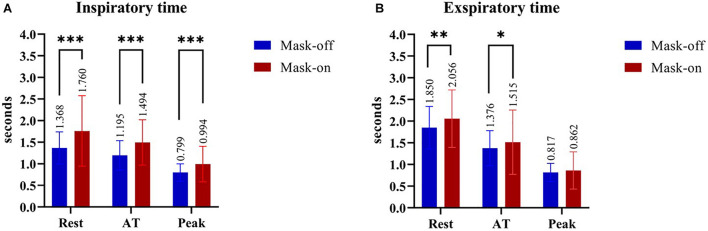
The comparison between mask-on and mask-off condition in the inspiratory time **(A)** and expiratory time **(B)** during different stages of the CPET, respectively. *P ≤ 0.05; ** *P* ≤ 0.01; ***P ≤ 0.001. AT, anaerobic threshold; CPET, cardiopulmonary exercise test.

The results of lung ventilation response parameters showed that at different stages of CPET with mask-on condition, Ti and Te were longer than mask-off (*p* < 0.05). The differences of Ti between mask-on and mask-off at rest, AT and peak period were 0.39 ± 0.76 s (*p* < 0.001), 0.30 ± 0.37 s (*p* < 0.001) and 0.20 ± 0.27 s (*p* < 0.001), respectively. The differences of Te between mask-on and mask-off at rest, AT and peak period were 0.21 ± 0.64 s (*p* = 0.008), 0.14 ± 0.47 s (*p* = 0.016) and 0.05 ± 0.27 s (*p* = 0.164), respectively (Figure 3).

Respiratory frequency and $\dot{\text{V}}$_*E*_ of mask-on were lower than mask-off at each stage of CPET (*p* < 0.05), especially at peak exercise (Rf_*peak*_: 33.8 ± 7.98 vs. 37.91 ± 6.72 b/min, *p* < 0.001;$\dot{\text{V}}$_*Epeak*_: 55.07 ± 17.28 vs. 66.46 ± 17.93 l/min, *p* < 0.001), and V_*T*_ was significantly lower than mask-off just at peak exercise (1.66 ± 0.45 vs. 1.79 ± 0.5 l, *p* < 0.001). BR% of mask-on was higher than mask-off at all stages of CPET (*p* < 0.001). There was no significant difference between mask-off and mask-on in S_*P*_O_2_ (*p* > 0.05) (Table 4).

**TABLE 4:** The comparison of pulmonary ventilation parameters between mask-on and mask-off condition.

	Mask-off	Mask-on	Cohen’s *d* effect size	*P* value
**$\dot{\text{V}}$_*E*_ (l/min)**				
Rest	10.7 ± 3.07	8.84 ± 2.94	0.62	**<0.001**
Warm up	15.8 ± 2.70	13.1 ± 3.64	0.82	**<0.001**
AT	29.5 ± 6.56	25.8 ± 8.13	0.50	**<0.001**
Peak	66.5 ± 17.9	55.1 ± 17.3	0.65	**<0.001**
**V_*T*_ (l)**				
Rest	0.59 ± 0.20	0.57 ± 0.27	0.08	0.083
Warm up	0.79 ± 0.28	0.74 ± 0.37	0.15	**0.017**
AT	1.28 ± 0.41	1.27 ± 0.49	0.02	0.388
Peak	1.79 ± 0.50	1.66 ± 0.45	0.27	**<0.001**
**BR (%)**				
Rest	91.3 ± 2.26	92.8 ± 2.37	0.64	**<0.001**
Warm up	87.3 ± 2.64	89.4 ± 2.87	0.76	**<0.001**
AT	76.7 ± 5.02	79.7 ± 5.43	0.58	**<0.001**
Peak	48.4 ± 10.9	57.0 ± 11.3	0.77	**<0.001**
**Rf (b/min)**				
Rest	19.1 ± 4.51	16.5 ± 4.59	0.56	**<0.001**
Warm up	21.4 ± 5.18	19.3 ± 5.52	0.38	**0.001**
AT	24.3 ± 5.65	21.7 ± 6.38	0.43	**<0.001**
Peak	37.9 ± 6.72	33.8 ± 7.98	0.55	**<0.001**
**SpO_2_ %**				
Rest	94.9 ± 20.6	97.9 ± 12.1	0.17	0.161
Warm up	95.8 ± 17.0	97.5 ± 12.2	0.11	0.144
AT	96.9 ± 12.3	96.8 ± 8.51	0.01	0.094
Peak	95.3 ± 8.72	95.3 ± 9.25	0.001	0.564
**PetO_2_ (mmHg)**				
Rest	108.9 ± 10.9	108.9 ± 6.09	0.005	0.266
Warm up	107.6 ± 9.68	105.1 ± 11.0	0.24	**<0.001**
AT	105.1 ± 8.59	103.2 ± 9.38	0.21	**0.002**
Peak	115.7 ± 9.75	112.2 ± 11.0	0.33	**<0.001**
**PetCO_2_ (mmHg)**				
Rest	34.2 ± 10.3	34.9 ± 8.03	0.08	**0.040**
Warm up	36.5 ± 8.85	37.4 ± 9.64	0.10	**0.027**
AT	42.2 ± 7.86	43.6 ± 8.40	0.17	**0.001**
Peak	38.8 ± 9.70	41.6 ± 10.2	0.28	**<0.001**
**$\dot{\text{V}}$_*E*_/$\dot{\text{V}}$O_2_**				
Rest	33.2 ± 5.49	32.6 ± 6.16	0.11	0.350
Warm up	30.3 ± 3.67	28.2 ± 4.93	0.47	**<0.001**
AT	27.4 ± 3.38	26.2 ± 4.04	0.33	**0.001**
Peak	39.6 ± 5.80	36.4 ± 6.82	0.51	**<0.001**
**$\dot{\text{V}}$_*E*_/$\dot{\text{V}}$CO_2_**				
Rest	38.8 ± 6.17	37.4 ± 7.24	0.21	**0.049**
Warm up	34.5 ± 4.46	32.6 ± 5.86	0.36	**<0.001**
AT	27.9 ± 3.34	26.9 ± 4.04	0.27	**0.002**
Peak	30.3 ± 3.87	28.8 ± 4.68	0.34	**<0.001**
$\dot{\text{V}}$_*E*_/$\dot{\text{V}}$CO_2_ slope	26.4 ± 3.38	26.0 ± 4.49	0.08	0.512

*The values were shown in mean ± standard deviation. Significance level was set at *P* < 0.05. $\dot{\text{V}}$_*E*_, minute ventilation; V_*T*_, tidal volume; BR, breathing reserve; Rf, breathing frequency; SpO_2_, percutaneous oxygen saturation; PetO_2_, end-tidal oxygen partial pressure; PetCO_2_, end-tidal carbon dioxide partial pressure; $\dot{\text{V}}$CO_2_, carbon dioxide production; $\dot{\text{V}}$CO_2_, carbon dioxide output; AT, anaerobic threshold. Significant results are indicated in bold.*

The result of pulmonary ventilation efficiency parameters showed that there were no significant differences in P_*et*_O_2_ and $\dot{\text{V}}$_*E*_/$\dot{\text{V}}$O_2_ between mask-off and mask-on during the rest period (*p* > 0.05), but PetO_2_ and $\dot{\text{V}}$_*E*_/$\dot{\text{V}}$O_2_ of mask-on during warm-up period to peak exercise period were lower than mask-off (*p* < 0.05). At all stages of CPET, P_*et*_CO_2_ of mask-on was higher than mask-off and $\dot{\text{V}}$_*E*_/$\dot{\text{V}}$CO_2_ was lower than mask-off (*p* < 0.05), but there was no significant difference in $\dot{\text{V}}$_*E*_/$\dot{\text{V}}$CO_2_ slope between them (*p* > 0.05) (Table 4).

### Cardiovascular Reaction Parameters

The result of cardiovascular response parameters showed that $\dot{\text{V}}$O_2_/HR, OUES, and △$\dot{\text{V}}$O_2_/△W of mask-on was significantly lower than mask-off ($\dot{\text{V}}$O_2__*peak*_/HR: 8.82 ± 2.6 vs. 9.51 ± 2.48 ml/beat, *p*< 0.001; OUES: 1641.2 ± 449.5 vs. 1914.4 ± 498.3 ml/min/l/min, *p* < 0.001; △$\dot{\text{V}}$O_2_/△W: 8.04 ± 1.03 vs. 8.55 ± 0.9 mlO_2_/W, *p* = 0.002), while HR, HRR, and systolic BP were significantly different at peak exercise (*p* < 0.05), and diastolic BP had no significant difference at different stages of CPET (*p* > 0.05) (Table 5).

**TABLE 5:** The comparison of cardiovascular reaction parameters between mask-on and mask-off condition.

	Mask-off	Mask-on	Cohen’s *d* effect size	*P* value
**HR (bpm)**				
Rest	82.6 ± 11.5	82.0 ± 9.38	0.05	0.691
AT	127.8 ± 15.3	126.7 ± 16.2	0.13	0.557
Peak	171.0 ± 13.7	165.8 ± 15.7	0.35	**<0.001**
HRR (bpm)	22.0 ± 12.9	26.2 ± 14.2	0.31	**0.006**
**$\dot{\text{V}}$O_2_/HR (ml/beat)**				
Rest	3.56 ± 1.42	2.96 ± 1.18	0.46	**<0.001**
AT	8.02 ± 1.90	7.39 ± 2.20	0.30	**<0.001**
Peak	9.51 ± 2.48	8.82 ± 2.60	0.27	**<0.001**
**Psyst (mmHg)**				
Rest	104.3 ± 26.2	109.2 ± 15.5	0.21	0.561
AT	127.0 ± 20.9	130.0 ± 22.9	0.14	0.262
Peak	162.6 ± 25.5	160.9 ± 30.7	0.06	**0.048**
**Pdiast (mmHg)**				
Rest	68.1 ± 18.1	72.2 ± 11.4	0.25	0.130
AT	73.5 ± 12.6	74.2 ± 13.5	0.06	0.637
Peak	88.0 ± 20.6	87.0 ± 20.4	0.05	0.747
**OUES (ml/ml/l/min)**	1914.4 ± 498.3	1641.2 ± 449.5	0.57	**<0.001**
**△$\dot{\text{V}}$O_2_/△W (mlO_2_/Watt)**	8.55 ± 0.90	8.04 ± 1.03	0.52	**0.002**

*The values were shown in mean ± standard deviation. Significance level was set at *P* < 0.05. HR, heart rate; HRR, heart rate reserve; $\dot{\text{V}}$O_2_, oxygen uptake; Psyst, systolic pressure; Pdiast, diastolic pressure; OUES, oxygen uptake efficiency slope; AT, anaerobic threshold. Significant results are indicated in bold.*

### Adverse Event Reported

No adverse events such as hypoxemia, myocardial ischemia, arrhythmia, and hypoperfusion occurred in any of the subjects in this study.

## Discussion

Mask could effectively prevent infectious respiratory diseases, and the role of the mask in COVID-19 has been confirmed (Dharmadhikari et al., 2012; Chu et al., 2020). This study selected surgical mask and investigated the physiological effects of masks on healthy people under exercise load through self-comparison. The result demonstrated that that mask had significant effects on cardiopulmonary function (including $\dot{\text{V}}$_*E*_, V_*T*_, Rf, BR%, $\dot{\text{V}}$CO_2_, P_*ET*_O_2_, P_*ET*_CO_2_, $\dot{\text{V}}$_*E*_/$\dot{\text{V}}$O_2_, $\dot{\text{V}}$_*E*_/$\dot{\text{V}}$CO_2_, $\dot{\text{V}}$O_2_/HR, OUES, and △$\dot{\text{V}}$O_2_/△W) and cardiopulmonary fitness or exercise endurance (such as $\dot{\text{V}}$O_2__*peak*_ and $\dot{\text{V}}$O_2__*peak*_/kg), and lung function was significantly affected.

First, surgical masks influenced the performance in CPET. This research suggested that the exercise test time and a maximum power of healthy subjects wearing masks were lower than those without masks, and the dyspnea index was increased. In the study on 31 adults (Driver et al., 2021) cloth face masks led to a 14% reduction in exercise time and attributed to perceived discomfort (such as feeling increasingly short of breath and claustrophobic at higher exercise intensities) associated with mask-wearing. Therefore, masks could affect the exercise performance and subjective feelings of healthy subjects.

Second, surgical masks had influences on lung function. Both this study and Mapelli’s (Mapelli et al., 2021) study found out that after wearing masks, Ti and Te increased since rest period, especially Ti, which indirectly reflected that masks could increase the inspiratory and expiratory resistance of the oronasal airway. The increase of facial temperature and humidity during exercise could also cause moisture and deformation of the mask, which could further increase respiratory resistance. Different types of masks cause different respiratory resistance, which led to the greater the respiratory resistance, the larger the dead space, and the greater the influence on the ventilation function and ventilation efficiency (Jones et al., 1971). Studies had shown that after wearing N95 filter masks, the inspiratory and expiratory resistance increased by 0.43 and 0.23 mmH_2_O, respectively (Roberge et al., 2010a). An animal study measured the maximum speed of six horses in the treadmill exercise test, and then exercised on the treadmill at the maximum speeds of 50, 75, and 100%, respectively with and without masks. The results showed that compared with those without masks, the difference of peak inspiratory pressure between trachea and pharynx increased negatively, peak expiratory pressure between trachea and pharynx increased positively, and Rf was lower (*p* < 0.05) (Holcombe et al., 1996). The increase of respiratory resistance prolonged the breathing time to meet the ventilation needs, which led to the slowdown of Rf after wearing a mask. Meanwhile, the increase of respiratory resistance and the slowdown of Rf led to the decrease of V_*T*_, showing insufficient ventilation, resulting in the decrease of $\dot{\text{V}}$_*E*_ and the increase of BR%. This study confirmed that Rf, V_*T*_, and $\dot{\text{V}}$_*E*_ decreased and BR% increased after wearing surgical masks, especially in the peak period of exercise. Seo et al. (2017) tested nine healthy men wearing masks with different inspiratory resistances, and the results also showed that Rf and $\dot{\text{V}}$_*E*_ decreased with the increase of inspiratory resistance. However, Roberge et al. studies20 healthy adult subjects walking on a flat plate at a speed of 5.6 km/h for 1 h with or without wearing surgical masks, and the results showed that wearing masks caused an increase in Rf by 1.6 beats/min (*p* = 0.02) (Roberge et al., 2012). It was also observed that the subjects walked at the same speed for 1 h while wearing N95 masks, and the Rf of the mask group increased by 1.4–2.4 beats/min (*p* < 0.05) compared with the control group (Kim et al., 2013). The exercise method used in these two studies was to walk on a flat plate at a constant speed, with limb muscles and even chest muscles participating in the exercise for a longer period. However, this study used cycle ergometer incremental exercise, with mainly lower limb muscles participating for a shorter time, so there were differences in breathing patterns between them, and individual differences of the subjects could also affect the results. In addition, in this study, although V_*T*_ and $\dot{\text{V}}$_*E*_ decreased after wearing surgical masks, they were all in the normal range, and SpO_2_ did not decrease during the whole exercise process, which did not cause the compensatory mechanism of the body. If the subjects exercised in high intensity for a longer time, it might cause compensatory acceleration of Rf (Qiu and Wang, 2012). According to the results of this study, the respiratory pattern change caused by wearing surgical masks was insufficient ventilation, which affected the ventilation function.

The increase of respiratory resistance after wearing masks would increase the work done by respiratory muscles and affected the gas exchange and ventilation efficiency. In this study, $\dot{\text{V}}$O_2_, $\dot{\text{V}}$CO_2_, PetO_2_, $\dot{\text{V}}$_*E*_/$\dot{\text{V}}$O_2_, and $\dot{\text{V}}$_*E*_/$\dot{\text{V}}$CO_2_ all decreased and PetCO_2_ increased after wearing surgical masks. The changes existed in the rest period or warm-up exercise and became more significant as exercise intensity increased. As inspiratory resistance increased, the inhaled oxygen concentration decreased, which led to the decrease of $\dot{\text{V}}$O_2_ and PetO_2_. $\dot{\text{V}}$_*E*_ decreased more significantly than $\dot{\text{V}}$O_2_, so $\dot{\text{V}}$_*E*_/$\dot{\text{V}}$O_2_ decreased. The study showed that $\dot{\text{V}}$_*E*_/$\dot{\text{V}}$O_2_ could be decreased by 12–31% with the increase of inspiratory resistance (Caretti and Whitley, 1998). In addition, the expiratory resistance increased and the dead space increased, resulting in a decrease in $\dot{\text{V}}$CO_2_ and a higher PaCO_2_, showing a relative carbon dioxide retention performance (Holcombe et al., 1996). Umutlu et al. (2021) also indicated that $\dot{\text{V}}$O_2_, $\dot{\text{V}}$CO_2_, and $\dot{\text{V}}$_*E*_ decreased significantly during aerobic exercise (*p* < 0.001). Because $\dot{\text{V}}$_*E*_ decreased more significantly than $\dot{\text{V}}$CO_2_, so the decrease in $\dot{\text{V}}$_*E*_/$\dot{\text{V}}$CO_2_ was well proved in this study. However, due to the limited discharge of carbon dioxide, the concentration of carbon dioxide in the mask and inhaled increased (Roberge et al., 2010b; Smith et al., 2013). Therefore, this study observed that PetCO_2_ was higher when wearing surgical masks than mask-off (*p* < 0.001). Roberge et al. (2012) also found out that after wearing masks, the percutaneous carbon dioxide partial pressure increased by 2.17 mmHg (*P* = 0.0006), and a similar change existed when exercising with N95 mask (Kim et al., 2013; Goh et al., 2019). Epstein et al. (2021) found PetCO_2_ increased with the increase of exercise load after wearing N95 masks, and it increased by 8 mmHg at peak exercise. It could be seen that wearing masks presented pathophysiological changes similar to COPD, which would reduce ventilation efficiency, especially during high-intensity exercise. In this study, no hypoxemia occurred in healthy subjects, which was related to the strong compensatory ability of healthy people. However, for patients with respiratory diseases, such as COPD or heart failure, the above physiological changes might aggravate the condition of the patient (Hopkins et al., 2021). Therefore, such patients were required to be fully evaluated before exercising with masks.

Third, surgical masks influenced cardiopulmonary fitness and exercise endurance. Cardiopulmonary fitness has been listed as the fifth vital sign by AHA, which not only reflected exercise endurance but also was an effective index for disease occurrence risk and death risk (Ross et al., 2016). Maximum $\dot{\text{V}}$O_2_ was often used to evaluate cardiopulmonary fitness. The walking test (6 min) was a simple and easy method to evaluate cardiopulmonary fitness and exercise endurance. Person et al. (2018) randomly divided 44 healthy people into a mask-on and a mask-off group and conducted a 6 min walking test, respectively. The results showed that the 6 min walk distance, HR, and SpO_2_ did not change significantly, and only the dyspnea index increased significantly. The 6 min walking test was a kind of sub-maximal exercise test, which was not accurate enough to fully reflect the cardiopulmonary function and metabolism, however, CPET was more accurate. Through CPET, this study found out that $\dot{\text{V}}$O_2_ had decreased since the rest period, and $\dot{\text{V}}$O_2__*peak*_ was decreased by about 11% (*p* < 0.001). After wearing FFP2/N95 mask, $\dot{\text{V}}$O_2__*peak*_ could decrease by 13% (Fikenzer et al., 2020). In the study of Driver et al., cloth face masks led to a 29% decrease in $\dot{\text{V}}$O_2__*max*_ (*p* < 0.001) (Driver et al., 2021). Dressendorfer et al. (1977) showed that $\dot{\text{V}}$O_2__*peak*_ after wearing masks was 10–15% lower than the reference range, accompanied by a slight decrease in $\dot{\text{V}}$CO_2_ and RER. If the mask was removed before the peak exercise, both $\dot{\text{V}}$O_2_ and $\dot{\text{V}}$_*E*_ could be significantly increased, and exercise could be maintained for at least 1 min. The increase of respiratory resistance decreased the concentration of inhaled oxygen, caused the respiratory muscles to do extra work, and increased oxygen consumption, which led to the changes in the above-mentioned lung function. In addition, the heat and humidity of the mask increased during exercise (Roberge et al., 2012; Scarano et al., 2020). All mentioned above could cause a decrease in $\dot{\text{V}}$O_2_, shorten the exercise time, and decreased the maximum power in exercise tests (Johnson, 2016). The AT was also an index reflecting the aerobic capacity of the body. A Japanese study of six young men showed that surgical masks did not affect the AT during treadmill exercise (Otsuka et al., 2020). In Egger’s research on athletes, surgical masks or N95 masks led to a reduction in $\dot{\text{V}}$O_2_max, but there was no significant difference in the AT, which might be related to the strong aerobic metabolism ability and cardiopulmonary fitness of athletes (Egger et al., 2021). However, the AT and $\dot{\text{V}}$O_2_@AT/$\dot{\text{V}}$O_2_max pre % were significantly different in this study, suggesting that AT appeared ahead of schedule. Relative carbon dioxide retention after wearing masks led to anaerobic metabolism ahead of schedule. Therefore, wearing masks would affect cardiopulmonary fitness and reduce exercise endurance. There might be safety risks for patients with cardiopulmonary disease, and such patients needed to be fully evaluated.

Fourth, surgical masks influenced cardiovascular function. In general, the blood flow is redistributed during exercise, and the blood flow of the myocardium increased to ensure the blood pumping function of the heart. The excitation of the sympathetic nerve led to the enhancement of cardiac systolic function, the increase of stroke volume (SV), HR, CO, and BP. After wearing masks, the airway resistance increased, the negative pressure of the chest increased when inhaling, and the blood flow increased as well, increasing cardiac preload (Cooke et al., 2006). On the other hand, the contraction of peripheral vessels and the increase of cardiac afterload during exercise could cause the compensatory increase of SV, HR, and CO, and at the same time increased the extra work of the heart, which led to the decrease of work efficiency and oxygen utilization capacity of the heart (Cheyne et al., 2020). Umutlu et al. (2021) conducted CPET and walk test on 14 sedentary volunteers (all on a treadmill), showing that HR systolic BP and diastolic BP increased significantly after wearing masks (*p* < 0.01). Lässing et al. (2020) studied the changes in cardiopulmonary function during constant power test with surgical masks and found that HR_*peak*_ increased significantly (*p* < 0.01), SV and CO increased slightly (*p* > 0.05), and arteriovenous oxygen difference (avDO_2_) decreased significantly (*p* = 0.02). This study suggested that the decrease of $\dot{\text{V}}$O_2_ after wearing masks was mainly related to the decrease of avDO_2_ ($\dot{\text{V}}$O_2_ = CO × avDO_2_), while the change of lung function caused by wearing a mask could lead to the decrease of avDO_2_; thus, the decrease of $\dot{\text{V}}$O_2_ was mainly related to the change of lung function. However, Fikenzer et al. (2020) adopted the step-by-step incremental protocol for CPET. The results showed that the HR_*peak*_ of exercise decreased when wearing surgical masks and FFP2/N95 masks, but it was more significant when wearing surgical masks (*p* < 0.05), and the SV and CO were slightly higher than those without masks (*p* > 0.05). Similarly, in this study, the HR_*peak*_ of healthy volunteers decreased when exercising with surgical masks (*p* < 0.05), which might be related to the different CPET protocols and the short exercise time of CPET. There might be an obvious compensatory increase in HR for long-term high-intensity exercise, which needed further study. In addition, surgical masks led to a significant decrease in $\dot{\text{V}}$O_2_/HR, OUES (ratio of $\dot{\text{V}}$O_2_ to the logarithm of ventilation volume), and △$\dot{\text{V}}$O_2_/△W in this study, which was mainly related to the decrease of $\dot{\text{V}}$O_2_, indicating that wearing masks caused the decrease of oxygen transport capacity of heart and oxygen utilization capacity of the body in healthy people.

To summarize, surgical masks had a certain influence on the heart function of healthy people during exercise, which was mainly due to the limitation of lung function. For patients with heart diseases, this influence might be enlarged due to the damage of heart compensatory function.

### Limitations

First, all the subjects included in this study were healthy people of low age, and the results could not reflect the influence of surgical masks on exercise cardiopulmonary function of middle-aged and elderly people and patients with cardiopulmonary disease. Second, this study did not combine blood gas analysis to accurately evaluate metabolism, which could more effectively reflect aerobic and anaerobic metabolism *in vivo* by measuring arterial oxygen partial pressure, carbon dioxide partial pressure, and lactic acid value.

## Conclusion

Wearing surgical masks during aerobic exercise showed certain negative impacts on cardiopulmonary function, especially during high-intensity exercise in healthy young subjects. These results provide an important recommendation for wearing a mask at a pandemic during exercises of varying intensity. Future research should focus on the response of wearing masks in patients with related cardiopulmonary diseases.

## Data Availability Statement

The original contributions presented in the study are included in the article/supplementary material, further inquiries can be directed to the corresponding authors.

## Ethics Statement

The studies involving human participants were reviewed and approved by the Research Ethics Committee Guangdong Provincial People’s Hospital, Guangdong Academy of Medical Sciences. The patients/participants provided their written informed consent to participate in this study.

## Author Contributions

GZ and ML assisted in the subject recruitment, data collection, data analysis, and writing. MZ, XC, JY, SZ, and AY assisted with the data collection and data analysis. YZ, QL, and JL assisted with the data analysis. LG and HO assisted with the study design, data analysis, and manuscript editing. All authors contributed to the article and approved the submitted version.

## Conflict of Interest

The authors declare that the research was conducted in the absence of any commercial or financial relationships that could be construed as a potential conflict of interest.

## Publisher’s Note

All claims expressed in this article are solely those of the authors and do not necessarily represent those of their affiliated organizations, or those of the publisher, the editors and the reviewers. Any product that may be evaluated in this article, or claim that may be made by its manufacturer, is not guaranteed or endorsed by the publisher.
